# 4*H*-Cyclo­penta­[*def*]phenanthren-4-one

**DOI:** 10.1107/S1600536809030633

**Published:** 2009-08-08

**Authors:** Wen-Sheng Jiang, Di Sun, Su-Yuan Xie, Rong-Bin Huang, Lan-Sun Zheng

**Affiliations:** a Department of Chemistry, College of Chemistry and Chemical Engineering, Xiamen University, Xiamen 361005, People’s Republic of China; b State Key Laboratory for Physical Chemistry of Solid Surfaces, Xiamen University, Xiamen 361005, People’s Republic of China

## Abstract

In the title compound, C_15_H_8_O, the asymmetric unit contains four independent mol­ecules and crystallizes with aromatic π–π stacking inter­actions[centroid–centroid distances = 3.5326 (18) Å].

## Related literature

The title compound (Muzart, 1987 ▶) can be readily obtained by oxidation of the corresponding hydro­carbon, 4*H*-cyclo­penta­[*def*]-phenanthrene, see: Yang & Harvey (1992 ▶). We recently obtained it in our low pressure premixed benzene–oxygen combustion system, see: Sun *et al.* (2008 ▶). For our work on the use of a variety of non-organic methods to generate and trap a family of chlorinated fullerene fragments and clusters, see: Huang *et al.* (1997 ▶); Peng *et al.* (2001 ▶); Tan *et al.* (2008 ▶); Xie *et al.* (2001 ▶, 2004 ▶); For a related structures, see: Peng *et al.* (2004 ▶). For the synthesis, see: Harvey *et al.* (1992 ▶).
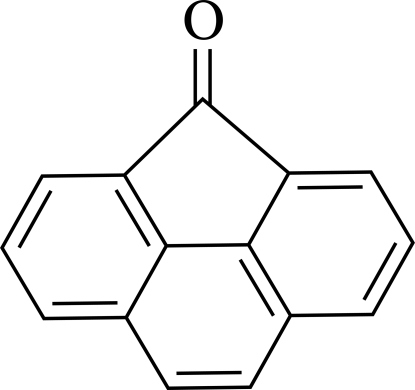


## Experimental

### 

#### Crystal data

C_15_H_8_O
*M* *_r_* = 204.1
Triclinic, 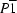

*a* = 7.7884 (5) Å
*b* = 15.1558 (8) Å
*c* = 17.0236 (10) Å
α = 100.588 (5)°
β = 101.065 (5)°
γ = 91.824 (5)°
*V* = 1933.8 (2) Å^3^
*Z* = 8
Mo *K*α radiation
μ = 0.09 mm^−1^
*T* = 293 K
0.45 × 0.22 × 0.20 mm

#### Data collection

Oxford Gemini S Ultra diffractometer
Absorption correction: multi-scan (*CrysAlis RED*; Oxford Diffraction, 2007 ▶) *T*_min_ = 0.962, *T*_max_ = 0.983
16146 measured reflections
6739 independent reflections
4205 reflections with *I* > 2σ(*I*)
*R*_int_ = 0.036

#### Refinement

*R*[*F* ^2^ > 2σ(*F* ^2^)] = 0.051
*wR*(*F* ^2^) = 0.129
*S* = 0.93
6739 reflections
577 parameters
30 restraints
H-atom parameters constrained
Δρ_max_ = 0.41 e Å^−3^
Δρ_min_ = −0.26 e Å^−3^

### 

Data collection: *CrysAlis CCD* (Oxford Diffraction, 2007 ▶); cell refinement: *CrysAlis RED* (Oxford Diffraction, 2007 ▶); data reduction: *CrysAlis RED*; program(s) used to solve structure: *SHELXS97* (Sheldrick, 2008 ▶); program(s) used to refine structure: *SHELXL97* (Sheldrick, 2008 ▶); molecular graphics: *SHELXL97*; software used to prepare material for publication: *SHELXL97* and *publCIF* (Westrip, 2009 ▶).

## Supplementary Material

Crystal structure: contains datablocks I, global. DOI: 10.1107/S1600536809030633/bx2227sup1.cif

Structure factors: contains datablocks I. DOI: 10.1107/S1600536809030633/bx2227Isup2.hkl

Additional supplementary materials:  crystallographic information; 3D view; checkCIF report

## Figures and Tables

**Fig. 1.**
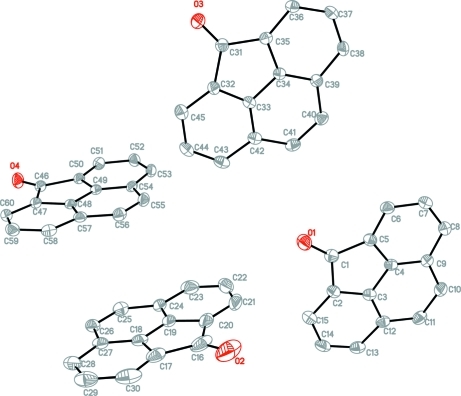
ORTEP plot of the compound. The thermal ellipsoids are drawn at 30% probability level, hydrogen atoms are omitted for clarity.

## supplementary crystallographic information

### Comment

The title compound (I) (Muzart,1987) can be readily obtained by oxidation of the corresponding hydrocarbon, 4*H*-cyclopenta[def]-phenanthrene (Yang & Harvey 1992), but its crystal structure determination has not been carried out yet. During the past decade, our group has used various non-organic methods, such as high-voltage electric discharge in liquid (Huang *et al.*, 1997), vaporized (Xie *et al.*, 2001, 2004) chloroform and CCl_4_ and solvothermal reaction (Peng *et al.*, 2001) to generate and trap a family of chlorinated fullerene fragments and clusters (Tan *et al.*, 2008). Recently in our low pressure premixed benzene-oxygen combustion system (Sun *et al.*, 2008), we obtained the title compound, C_15_H_8_O. The skeleton of title compound is similar to that of previously reported, C_15_Cl_8_O, (Peng *et al.*,2004). We report here the synthesis and crystal structure of the title compound, (I) (Figure 1), which was separated from the products of combustion process. Due to the aromatic nature of the molecule, the crystal packing of (I) is dominated by arene-arene supramolecular contacts and characterized by molecular stacks which are stabilized by offset face-to-face interactions.

### Experimental

The title compound, C_15_H_8_O, was prepared in low pressure premixed benzene-oxygen flames. The premixed flames conditions for the soot production as the following range: atom C/O ratio:1–2; combustion chamber pressure: 350 torr. The soot collected from the water-cooled coping was extracted with toluene using an ultrasonic bath under room temperature, the resulting nigger-brown solution separated and purified by multi-stage HPLC, finally we obtained one of fractions contained pure C_15_H_8_O. The red single crystals suitable for X-ray diffraction crystallized from toluene at room temperature only in one day. The product was analyzed by mass spectrometry. The molecular peak appeared at a mass/charge ratio of 204.

### Refinement

All H atoms were placed geometrically with C—H distances of 0.95 Å, N—H distances of 0.88Å and refined using a riding atom model with their isotropic displacement factors, J_iso_ fixed at 1.2 time the *U*_eq_ of the parent N and phenyl C atom, at 1.5 of methyl C atom.

### Crystal data

**Table:**

C_15_H_8_O	*Z* = 8
*M**_r_* = 204.1	*F*(000) = 848
Triclinic, *P*1	*D*_x_ = 1.403 Mg m^−^^3^
Hall symbol: -P 1	Mo *K*α radiation, λ = 0.71073 Å
*a* = 7.7884 (5) Å	Cell parameters from 4555 reflections
*b* = 15.1558 (8) Å	θ = 2.5–32.6°
*c* = 17.0236 (10) Å	µ = 0.09 mm^−^^1^
α = 100.588 (5)°	*T* = 293 K
β = 101.065 (5)°	Prism, red
γ = 91.824 (5)°	0.45 × 0.22 × 0.20 mm
*V* = 1933.8 (2) Å^3^	

### Data collection

**Table:**

Oxford Gemini S Ultra diffractometer	6739 independent reflections
Radiation source: fine-focus sealed tube	4205 reflections with *I* > 2σ(*I*)
graphite	*R*_int_ = 0.036
Detector resolution: 16.1903 pixels mm^-1^	θ_max_ = 25.0°, θ_min_ = 2.5°
ω scans	*h* = −9→8
Absorption correction: multi-scan (*CrysAlis RED*; Oxford Diffraction, 2007)	*k* = −18→18
*T*_min_ = 0.962, *T*_max_ = 0.983	*l* = −20→20
16146 measured reflections	

### Refinement

**Table:**

Refinement on *F*^2^	Primary atom site location: structure-invariant direct methods
Least-squares matrix: full	Secondary atom site location: difference Fourier map
*R*[*F*^2^ > 2σ(*F*^2^)] = 0.051	Hydrogen site location: inferred from neighbouring sites
*wR*(*F*^2^) = 0.129	H-atom parameters constrained
*S* = 0.93	*w* = 1/[σ^2^(*F*_o_^2^) + (0.0688*P*)^2^] where *P* = (*F*_o_^2^ + 2*F*_c_^2^)/3
6739 reflections	(Δ/σ)_max_ < 0.001
577 parameters	Δρ_max_ = 0.41 e Å^−^^3^
30 restraints	Δρ_min_ = −0.26 e Å^−^^3^

### Special details

**Table:**

Experimental. CrysAlis RED, Oxford Diffraction (2007) Ltd., Version 1.171.32.5 (release 08-05 CrysAlis171 .NET) (compiled May 8 ,13:10:02) Empirical absorption correction using spherical harmonics, implemented in SCALE3 ABSPACK scaling algorithm.
Geometry. All e.s.d.'s (except the e.s.d. in the dihedral angle between two l.s. planes) are estimated using the full covariance matrix. The cell e.s.d.'s are taken into account individually in the estimation of e.s.d.'s in distances, angles and torsion angles; correlations between e.s.d.'s in cell parameters are only used when they are defined by crystal symmetry. An approximate (isotropic) treatment of cell e.s.d.'s is used for estimating e.s.d.'s involving l.s. planes.
Refinement. Refinement of *F*^2^ against ALL reflections. The weighted *R*-factor *wR* and goodness of fit *S* are based on *F*^2^, conventional *R*-factors *R* are based on *F*, with *F* set to zero for negative *F*^2^. The threshold expression of *F*^2^ > σ(*F*^2^) is used only for calculating *R*-factors(gt) *etc*. and is not relevant to the choice of reflections for refinement. *R*-factors based on *F*^2^ are statistically about twice as large as those based on *F*, and *R*- factors based on ALL data will be even larger.

### Fractional atomic coordinates and isotropic or equivalent isotropic displacement parameters (Å^2^)

**Table:**

	*x*	*y*	*z*	*U*_iso_*/*U*_eq_	
C1	0.8063 (4)	0.33098 (18)	0.03517 (17)	0.0399 (7)	
C2	0.9978 (3)	0.36444 (17)	0.05461 (16)	0.0367 (7)	
C3	1.0372 (3)	0.37987 (16)	−0.01840 (16)	0.0348 (6)	
C4	0.8892 (3)	0.35928 (16)	−0.08247 (16)	0.0340 (6)	
C5	0.7456 (4)	0.32899 (17)	−0.05450 (16)	0.0356 (6)	
C6	0.5917 (4)	0.30514 (18)	−0.11001 (17)	0.0408 (7)	
H6A	0.4922	0.2844	−0.0943	0.049*	
C7	0.5879 (4)	0.31286 (19)	−0.19101 (17)	0.0450 (8)	
H7A	0.4833	0.2974	−0.2288	0.054*	
C8	0.7328 (4)	0.34248 (18)	−0.21725 (17)	0.0446 (7)	
H8A	0.7241	0.3466	−0.2717	0.053*	
C9	0.8934 (4)	0.36642 (17)	−0.16181 (16)	0.0377 (7)	
C10	1.0617 (4)	0.39708 (19)	−0.17468 (18)	0.0473 (8)	
H10A	1.0728	0.4031	−0.2269	0.057*	
C11	1.2049 (4)	0.41741 (19)	−0.11228 (18)	0.0468 (8)	
H11A	1.3102	0.4372	−0.1236	0.056*	
C12	1.1994 (4)	0.40946 (17)	−0.02998 (17)	0.0392 (7)	
C13	1.3329 (4)	0.42358 (18)	0.04128 (19)	0.0472 (8)	
H13A	1.4461	0.4426	0.0388	0.057*	
C14	1.2970 (4)	0.40944 (19)	0.11378 (19)	0.0491 (8)	
H14A	1.3873	0.4202	0.1596	0.059*	
C15	1.1294 (4)	0.37934 (19)	0.12227 (17)	0.0463 (8)	
H15A	1.1093	0.3699	0.1723	0.056*	
C16	0.6568 (4)	0.5839 (3)	0.4052 (2)	0.0694 (10)	
C17	0.6797 (4)	0.5821 (2)	0.4937 (2)	0.0525 (8)	
C18	0.7531 (3)	0.50091 (18)	0.50378 (18)	0.0410 (7)	
C19	0.7789 (3)	0.45052 (19)	0.42931 (16)	0.0388 (7)	
C20	0.7242 (4)	0.4942 (3)	0.3652 (2)	0.0632 (11)	
C21	0.7356 (5)	0.4504 (3)	0.2896 (2)	0.0766 (12)	
H21A	0.6995	0.4765	0.2444	0.092*	
C22	0.8009 (5)	0.3669 (3)	0.2807 (2)	0.0779 (12)	
H22A	0.8062	0.3375	0.2283	0.093*	
C23	0.8598 (4)	0.3236 (2)	0.3453 (2)	0.0635 (10)	
H23A	0.9054	0.2675	0.3364	0.076*	
C24	0.8480 (4)	0.3675 (2)	0.42488 (17)	0.0424 (7)	
C25	0.8946 (4)	0.3359 (2)	0.49970 (18)	0.0471 (8)	
H25A	0.9442	0.2808	0.4990	0.057*	
C26	0.8696 (4)	0.38269 (18)	0.57053 (18)	0.0438 (7)	
H26A	0.9007	0.3591	0.6176	0.053*	
C27	0.7939 (3)	0.47103 (18)	0.57593 (17)	0.0384 (7)	
C28	0.7557 (4)	0.52866 (19)	0.64403 (19)	0.0503 (8)	
H28A	0.7793	0.5124	0.6949	0.060*	
C29	0.6842 (4)	0.6086 (2)	0.6360 (2)	0.0628 (10)	
H29A	0.6600	0.6452	0.6822	0.075*	
C30	0.6458 (4)	0.6379 (2)	0.5620 (3)	0.0669 (11)	
H30A	0.5990	0.6931	0.5587	0.080*	
C31	−0.1975 (3)	0.08775 (17)	0.10259 (15)	0.0332 (6)	
C32	−0.0113 (3)	0.11963 (16)	0.14630 (15)	0.0316 (6)	
C33	0.0789 (3)	0.13636 (15)	0.08627 (14)	0.0292 (6)	
C34	−0.0332 (3)	0.11806 (15)	0.00853 (15)	0.0285 (6)	
C35	−0.2014 (3)	0.08791 (16)	0.01345 (15)	0.0302 (6)	
C36	−0.3248 (3)	0.06629 (17)	−0.05763 (15)	0.0342 (6)	
H36A	−0.4383	0.0453	−0.0576	0.041*	
C37	−0.2745 (4)	0.07684 (17)	−0.13048 (16)	0.0375 (7)	
H37A	−0.3575	0.0626	−0.1789	0.045*	
C38	−0.1079 (4)	0.10733 (17)	−0.13356 (15)	0.0374 (7)	
H38A	−0.0813	0.1137	−0.1833	0.045*	
C39	0.0232 (3)	0.12901 (16)	−0.06143 (15)	0.0315 (6)	
C40	0.2048 (4)	0.16059 (17)	−0.04878 (17)	0.0391 (7)	
H40A	0.2498	0.1699	−0.0936	0.047*	
C41	0.3131 (4)	0.17747 (18)	0.02610 (17)	0.0399 (7)	
H41A	0.4295	0.1973	0.0306	0.048*	
C42	0.2528 (3)	0.16546 (16)	0.09839 (16)	0.0335 (6)	
C43	0.3441 (4)	0.17931 (17)	0.18063 (16)	0.0390 (7)	
H43A	0.4622	0.1991	0.1941	0.047*	
C44	0.2574 (4)	0.16333 (17)	0.24065 (17)	0.0406 (7)	
H44A	0.3200	0.1729	0.2942	0.049*	
C45	0.0794 (4)	0.13330 (17)	0.22509 (16)	0.0388 (7)	
H45A	0.0251	0.1231	0.2670	0.047*	
C46	0.8208 (3)	−0.10522 (18)	0.52660 (16)	0.0344 (6)	
C47	0.7622 (3)	−0.01303 (17)	0.55685 (15)	0.0320 (6)	
C48	0.7321 (3)	0.02944 (17)	0.48979 (15)	0.0304 (6)	
C49	0.7657 (3)	−0.02706 (17)	0.41971 (15)	0.0309 (6)	
C50	0.8189 (3)	−0.10950 (17)	0.43687 (15)	0.0319 (6)	
C51	0.8496 (4)	−0.17342 (18)	0.37382 (16)	0.0388 (7)	
H51A	0.8842	−0.2297	0.3823	0.047*	
C52	0.8269 (4)	−0.15119 (19)	0.29552 (16)	0.0409 (7)	
H52A	0.8489	−0.1939	0.2525	0.049*	
C53	0.7739 (3)	−0.06923 (19)	0.28018 (16)	0.0386 (7)	
H53A	0.7601	−0.0576	0.2276	0.046*	
C54	0.7405 (3)	−0.00288 (17)	0.34402 (15)	0.0328 (6)	
C55	0.6794 (4)	0.08598 (18)	0.34298 (17)	0.0399 (7)	
H55A	0.6614	0.1068	0.2941	0.048*	
C56	0.6472 (4)	0.14037 (18)	0.41073 (17)	0.0394 (7)	
H56A	0.6086	0.1971	0.4065	0.047*	
C57	0.6706 (3)	0.11364 (17)	0.48878 (16)	0.0341 (6)	
C58	0.6382 (3)	0.15905 (18)	0.56446 (17)	0.0403 (7)	
H58A	0.5969	0.2163	0.5688	0.048*	
C59	0.6678 (4)	0.11875 (18)	0.63199 (17)	0.0424 (7)	
H59A	0.6461	0.1500	0.6811	0.051*	
C60	0.7296 (3)	0.03219 (18)	0.62916 (16)	0.0378 (7)	
H60A	0.7478	0.0064	0.6753	0.045*	
O1	0.7197 (3)	0.30949 (15)	0.08162 (12)	0.0563 (6)	
O2	0.5989 (4)	0.64120 (19)	0.37039 (19)	0.1032 (10)	
O3	−0.3173 (2)	0.06667 (13)	0.13308 (11)	0.0466 (5)	
O4	0.8593 (3)	−0.16354 (13)	0.56584 (11)	0.0487 (5)	

### Atomic displacement parameters (Å^2^)

**Table:**

	*U*^11^	*U*^22^	*U*^33^	*U*^12^	*U*^13^	*U*^23^
C1	0.0362 (17)	0.0442 (17)	0.0390 (17)	0.0025 (13)	0.0127 (14)	0.0018 (13)
C2	0.0364 (17)	0.0351 (15)	0.0360 (16)	0.0059 (12)	0.0061 (13)	0.0012 (12)
C3	0.0315 (16)	0.0299 (15)	0.0414 (16)	0.0041 (12)	0.0067 (13)	0.0029 (12)
C4	0.0317 (16)	0.0279 (14)	0.0395 (16)	0.0022 (11)	0.0050 (13)	0.0016 (11)
C5	0.0327 (16)	0.0358 (15)	0.0369 (16)	0.0027 (12)	0.0064 (13)	0.0039 (12)
C6	0.0332 (17)	0.0438 (17)	0.0443 (18)	−0.0038 (13)	0.0084 (13)	0.0060 (13)
C7	0.0393 (18)	0.0504 (18)	0.0409 (18)	−0.0052 (14)	−0.0009 (14)	0.0084 (13)
C8	0.049 (2)	0.0484 (18)	0.0358 (16)	−0.0025 (14)	0.0065 (14)	0.0098 (13)
C9	0.0369 (17)	0.0385 (16)	0.0380 (17)	−0.0011 (13)	0.0072 (13)	0.0091 (12)
C10	0.0449 (19)	0.0537 (19)	0.0469 (18)	−0.0029 (15)	0.0115 (15)	0.0173 (14)
C11	0.0386 (18)	0.0475 (18)	0.059 (2)	−0.0019 (14)	0.0156 (15)	0.0165 (15)
C12	0.0345 (17)	0.0302 (15)	0.0514 (18)	0.0013 (12)	0.0064 (14)	0.0066 (13)
C13	0.0332 (17)	0.0432 (18)	0.061 (2)	−0.0038 (13)	0.0040 (15)	0.0066 (14)
C14	0.0395 (19)	0.0480 (18)	0.049 (2)	−0.0028 (14)	−0.0082 (15)	0.0019 (14)
C15	0.0435 (19)	0.0505 (18)	0.0395 (17)	0.0011 (14)	0.0025 (14)	0.0011 (14)
C16	0.0469 (19)	0.070 (2)	0.091 (2)	−0.0149 (16)	−0.0093 (17)	0.0422 (19)
C17	0.0331 (16)	0.0526 (18)	0.070 (2)	−0.0078 (14)	0.0007 (14)	0.0199 (16)
C18	0.0284 (16)	0.0365 (16)	0.055 (2)	−0.0100 (13)	0.0022 (13)	0.0089 (14)
C19	0.0296 (16)	0.0485 (18)	0.0372 (17)	−0.0114 (14)	0.0009 (13)	0.0139 (14)
C20	0.039 (2)	0.092 (3)	0.057 (2)	−0.0307 (19)	−0.0119 (16)	0.038 (2)
C21	0.065 (2)	0.105 (2)	0.060 (2)	−0.0390 (19)	−0.0037 (16)	0.0431 (19)
C22	0.073 (2)	0.107 (3)	0.0495 (19)	−0.042 (2)	0.0203 (17)	0.0042 (19)
C23	0.054 (2)	0.066 (2)	0.068 (2)	−0.0193 (17)	0.0235 (18)	−0.0038 (18)
C24	0.0345 (17)	0.054 (2)	0.0374 (17)	−0.0143 (14)	0.0069 (13)	0.0102 (14)
C25	0.0442 (19)	0.0440 (18)	0.053 (2)	−0.0002 (14)	0.0093 (15)	0.0099 (15)
C26	0.0389 (18)	0.0476 (18)	0.0422 (18)	−0.0092 (14)	0.0095 (14)	0.0023 (14)
C27	0.0301 (16)	0.0368 (16)	0.0446 (18)	−0.0074 (12)	0.0055 (13)	0.0023 (13)
C28	0.0458 (19)	0.0451 (19)	0.054 (2)	−0.0080 (15)	0.0112 (15)	−0.0041 (15)
C29	0.049 (2)	0.046 (2)	0.087 (3)	−0.0020 (16)	0.0202 (19)	−0.0094 (19)
C30	0.035 (2)	0.0394 (19)	0.120 (3)	0.0013 (15)	0.010 (2)	0.007 (2)
C31	0.0328 (16)	0.0344 (15)	0.0330 (15)	0.0025 (12)	0.0090 (12)	0.0054 (11)
C32	0.0340 (16)	0.0305 (14)	0.0300 (15)	0.0039 (11)	0.0050 (12)	0.0065 (11)
C33	0.0284 (15)	0.0269 (14)	0.0297 (14)	0.0041 (11)	0.0020 (12)	0.0023 (11)
C34	0.0302 (15)	0.0247 (13)	0.0302 (14)	0.0034 (11)	0.0061 (11)	0.0041 (11)
C35	0.0274 (15)	0.0286 (14)	0.0349 (15)	0.0013 (11)	0.0061 (12)	0.0070 (11)
C36	0.0262 (15)	0.0379 (16)	0.0360 (16)	−0.0007 (12)	0.0028 (12)	0.0052 (12)
C37	0.0373 (17)	0.0412 (16)	0.0287 (15)	0.0002 (13)	−0.0014 (12)	0.0019 (12)
C38	0.0453 (18)	0.0425 (16)	0.0261 (15)	0.0035 (13)	0.0098 (13)	0.0080 (12)
C39	0.0327 (16)	0.0316 (14)	0.0305 (15)	0.0037 (12)	0.0075 (12)	0.0051 (11)
C40	0.0388 (18)	0.0411 (16)	0.0399 (17)	−0.0008 (13)	0.0150 (14)	0.0074 (13)
C41	0.0305 (16)	0.0422 (16)	0.0470 (18)	−0.0030 (13)	0.0116 (13)	0.0057 (13)
C42	0.0308 (16)	0.0301 (14)	0.0371 (16)	0.0034 (12)	0.0047 (12)	0.0020 (11)
C43	0.0313 (16)	0.0341 (16)	0.0444 (17)	0.0037 (12)	−0.0037 (13)	0.0009 (13)
C44	0.0431 (18)	0.0409 (16)	0.0312 (16)	0.0054 (13)	−0.0044 (13)	0.0022 (12)
C45	0.0466 (19)	0.0376 (16)	0.0311 (16)	0.0080 (13)	0.0060 (13)	0.0053 (12)
C46	0.0309 (16)	0.0383 (16)	0.0346 (15)	−0.0021 (12)	0.0033 (12)	0.0129 (13)
C47	0.0284 (15)	0.0359 (15)	0.0317 (15)	−0.0029 (12)	0.0036 (11)	0.0099 (12)
C48	0.0223 (14)	0.0333 (15)	0.0336 (15)	−0.0038 (11)	0.0010 (11)	0.0071 (12)
C49	0.0229 (14)	0.0376 (16)	0.0314 (15)	−0.0042 (11)	0.0037 (11)	0.0077 (12)
C50	0.0275 (15)	0.0352 (15)	0.0337 (15)	−0.0006 (12)	0.0050 (12)	0.0100 (12)
C51	0.0393 (17)	0.0372 (16)	0.0417 (17)	0.0037 (13)	0.0095 (13)	0.0108 (13)
C52	0.0425 (18)	0.0468 (18)	0.0336 (16)	0.0032 (14)	0.0099 (13)	0.0057 (13)
C53	0.0366 (17)	0.0522 (18)	0.0302 (15)	0.0009 (13)	0.0088 (12)	0.0138 (13)
C54	0.0272 (15)	0.0372 (16)	0.0363 (16)	−0.0027 (12)	0.0060 (12)	0.0143 (12)
C55	0.0374 (17)	0.0474 (18)	0.0388 (17)	0.0022 (13)	0.0047 (13)	0.0213 (14)
C56	0.0374 (17)	0.0365 (16)	0.0455 (18)	0.0029 (13)	0.0054 (13)	0.0139 (13)
C57	0.0247 (15)	0.0360 (16)	0.0396 (16)	−0.0039 (12)	0.0015 (12)	0.0085 (12)
C58	0.0345 (17)	0.0334 (16)	0.0509 (19)	−0.0022 (13)	0.0054 (14)	0.0068 (13)
C59	0.0422 (18)	0.0417 (17)	0.0400 (17)	−0.0007 (13)	0.0101 (13)	−0.0016 (13)
C60	0.0371 (17)	0.0458 (18)	0.0303 (15)	−0.0037 (13)	0.0046 (12)	0.0098 (12)
O1	0.0450 (13)	0.0841 (16)	0.0400 (12)	−0.0058 (11)	0.0129 (10)	0.0094 (11)
O2	0.0798 (18)	0.0976 (19)	0.133 (2)	−0.0129 (14)	−0.0255 (15)	0.0757 (17)
O3	0.0400 (12)	0.0637 (13)	0.0390 (11)	−0.0042 (10)	0.0136 (9)	0.0132 (9)
O4	0.0678 (15)	0.0444 (12)	0.0379 (12)	0.0088 (10)	0.0101 (10)	0.0178 (10)

### Geometric parameters (Å, °)

**Table:**

C1—O1	1.213 (3)	C31—O3	1.213 (3)
C1—C5	1.503 (4)	C31—C35	1.512 (4)
C1—C2	1.514 (4)	C31—C32	1.516 (4)
C2—C15	1.366 (4)	C32—C45	1.366 (3)
C2—C3	1.393 (4)	C32—C33	1.400 (3)
C3—C12	1.389 (4)	C33—C42	1.376 (3)
C3—C4	1.409 (4)	C33—C34	1.413 (3)
C4—C9	1.381 (4)	C34—C39	1.381 (3)
C4—C5	1.393 (4)	C34—C35	1.396 (3)
C5—C6	1.368 (4)	C35—C36	1.372 (3)
C6—C7	1.400 (4)	C36—C37	1.405 (4)
C6—H6A	0.9300	C36—H36A	0.9300
C7—C8	1.381 (4)	C37—C38	1.377 (4)
C7—H7A	0.9300	C37—H37A	0.9300
C8—C9	1.407 (4)	C38—C39	1.417 (3)
C8—H8A	0.9300	C38—H38A	0.9300
C9—C10	1.446 (4)	C39—C40	1.441 (3)
C10—C11	1.368 (4)	C40—C41	1.363 (4)
C10—H10A	0.9300	C40—H40A	0.9300
C11—C12	1.437 (4)	C41—C42	1.438 (4)
C11—H11A	0.9300	C41—H41A	0.9300
C12—C13	1.416 (4)	C42—C43	1.418 (4)
C13—C14	1.369 (4)	C43—C44	1.380 (4)
C13—H13A	0.9300	C43—H43A	0.9300
C14—C15	1.411 (4)	C44—C45	1.405 (4)
C14—H14A	0.9300	C44—H44A	0.9300
C15—H15A	0.9300	C45—H45A	0.9300
C16—O2	1.191 (4)	C46—O4	1.214 (3)
C16—C17	1.488 (5)	C46—C47	1.514 (4)
C16—C20	1.559 (6)	C46—C50	1.514 (4)
C17—C30	1.380 (5)	C47—C60	1.368 (4)
C17—C18	1.399 (4)	C47—C48	1.396 (3)
C18—C27	1.371 (4)	C48—C57	1.379 (4)
C18—C19	1.408 (4)	C48—C49	1.409 (4)
C19—C24	1.379 (4)	C49—C54	1.383 (3)
C19—C20	1.384 (4)	C49—C50	1.392 (4)
C20—C21	1.356 (5)	C50—C51	1.371 (4)
C21—C22	1.372 (5)	C51—C52	1.414 (4)
C21—H21A	0.9300	C51—H51A	0.9300
C22—C23	1.393 (5)	C52—C53	1.376 (4)
C22—H22A	0.9300	C52—H52A	0.9300
C23—C24	1.417 (4)	C53—C54	1.408 (4)
C23—H23A	0.9300	C53—H53A	0.9300
C24—C25	1.429 (4)	C54—C55	1.445 (4)
C25—C26	1.334 (4)	C55—C56	1.359 (4)
C25—H25A	0.9300	C55—H55A	0.9300
C26—C27	1.475 (4)	C56—C57	1.439 (4)
C26—H26A	0.9300	C56—H56A	0.9300
C27—C28	1.404 (4)	C57—C58	1.417 (4)
C28—C29	1.368 (4)	C58—C59	1.383 (4)
C28—H28A	0.9300	C58—H58A	0.9300
C29—C30	1.394 (5)	C59—C60	1.407 (4)
C29—H29A	0.9300	C59—H59A	0.9300
C30—H30A	0.9300	C60—H60A	0.9300
			
O1—C1—C5	126.9 (3)	O3—C31—C35	127.6 (2)
O1—C1—C2	127.6 (3)	O3—C31—C32	127.0 (2)
C5—C1—C2	105.4 (2)	C35—C31—C32	105.5 (2)
C15—C2—C3	118.1 (3)	C45—C32—C33	118.0 (2)
C15—C2—C1	135.8 (3)	C45—C32—C31	135.6 (2)
C3—C2—C1	106.1 (2)	C33—C32—C31	106.4 (2)
C12—C3—C2	126.4 (3)	C42—C33—C32	126.4 (2)
C12—C3—C4	122.5 (3)	C42—C33—C34	123.0 (2)
C2—C3—C4	111.1 (2)	C32—C33—C34	110.7 (2)
C9—C4—C5	126.4 (2)	C39—C34—C35	126.5 (2)
C9—C4—C3	122.9 (2)	C39—C34—C33	122.4 (2)
C5—C4—C3	110.6 (2)	C35—C34—C33	111.1 (2)
C6—C5—C4	117.7 (2)	C36—C35—C34	117.8 (2)
C6—C5—C1	135.6 (2)	C36—C35—C31	135.8 (2)
C4—C5—C1	106.7 (2)	C34—C35—C31	106.4 (2)
C5—C6—C7	118.2 (2)	C35—C36—C37	118.0 (2)
C5—C6—H6A	120.9	C35—C36—H36A	121.0
C7—C6—H6A	120.9	C37—C36—H36A	121.0
C8—C7—C6	122.9 (3)	C38—C37—C36	123.0 (2)
C8—C7—H7A	118.5	C38—C37—H37A	118.5
C6—C7—H7A	118.5	C36—C37—H37A	118.5
C7—C8—C9	120.2 (3)	C37—C38—C39	120.4 (2)
C7—C8—H8A	119.9	C37—C38—H38A	119.8
C9—C8—H8A	119.9	C39—C38—H38A	119.8
C4—C9—C8	114.6 (2)	C34—C39—C38	114.3 (2)
C4—C9—C10	115.0 (2)	C34—C39—C40	114.7 (2)
C8—C9—C10	130.4 (3)	C38—C39—C40	131.0 (2)
C11—C10—C9	121.9 (3)	C41—C40—C39	122.8 (2)
C11—C10—H10A	119.1	C41—C40—H40A	118.6
C9—C10—H10A	119.1	C39—C40—H40A	118.6
C10—C11—C12	122.7 (3)	C40—C41—C42	121.9 (2)
C10—C11—H11A	118.7	C40—C41—H41A	119.1
C12—C11—H11A	118.7	C42—C41—H41A	119.1
C3—C12—C13	114.0 (3)	C33—C42—C43	114.6 (2)
C3—C12—C11	114.9 (2)	C33—C42—C41	115.2 (2)
C13—C12—C11	131.0 (3)	C43—C42—C41	130.2 (3)
C14—C13—C12	120.6 (3)	C44—C43—C42	119.9 (3)
C14—C13—H13A	119.7	C44—C43—H43A	120.1
C12—C13—H13A	119.7	C42—C43—H43A	120.1
C13—C14—C15	123.1 (3)	C43—C44—C45	123.4 (2)
C13—C14—H14A	118.5	C43—C44—H44A	118.3
C15—C14—H14A	118.5	C45—C44—H44A	118.3
C2—C15—C14	117.8 (3)	C32—C45—C44	117.8 (3)
C2—C15—H15A	121.1	C32—C45—H45A	121.1
C14—C15—H15A	121.1	C44—C45—H45A	121.1
O2—C16—C17	128.2 (4)	O4—C46—C47	127.3 (2)
O2—C16—C20	125.8 (4)	O4—C46—C50	127.4 (2)
C17—C16—C20	106.1 (3)	C47—C46—C50	105.3 (2)
C30—C17—C18	117.8 (3)	C60—C47—C48	118.0 (2)
C30—C17—C16	136.2 (4)	C60—C47—C46	135.6 (2)
C18—C17—C16	105.9 (3)	C48—C47—C46	106.3 (2)
C27—C18—C17	125.6 (3)	C57—C48—C47	126.2 (2)
C27—C18—C19	122.6 (3)	C57—C48—C49	122.8 (2)
C17—C18—C19	111.7 (3)	C47—C48—C49	110.9 (2)
C24—C19—C20	126.9 (3)	C54—C49—C50	125.9 (2)
C24—C19—C18	121.5 (3)	C54—C49—C48	123.0 (3)
C20—C19—C18	111.6 (3)	C50—C49—C48	111.0 (2)
C21—C20—C19	117.0 (4)	C51—C50—C49	118.0 (2)
C21—C20—C16	138.3 (3)	C51—C50—C46	135.5 (3)
C19—C20—C16	104.7 (3)	C49—C50—C46	106.4 (2)
C20—C21—C22	119.2 (3)	C50—C51—C52	117.9 (3)
C20—C21—H21A	120.4	C50—C51—H51A	121.0
C22—C21—H21A	120.4	C52—C51—H51A	121.0
C21—C22—C23	124.0 (4)	C53—C52—C51	122.9 (3)
C21—C22—H22A	118.0	C53—C52—H52A	118.5
C23—C22—H22A	118.0	C51—C52—H52A	118.5
C22—C23—C24	118.1 (3)	C52—C53—C54	120.0 (2)
C22—C23—H23A	121.0	C52—C53—H53A	120.0
C24—C23—H23A	121.0	C54—C53—H53A	120.0
C19—C24—C23	114.9 (3)	C49—C54—C53	115.2 (2)
C19—C24—C25	116.7 (3)	C49—C54—C55	114.4 (2)
C23—C24—C25	128.4 (3)	C53—C54—C55	130.4 (2)
C26—C25—C24	122.3 (3)	C56—C55—C54	122.5 (2)
C26—C25—H25A	118.8	C56—C55—H55A	118.8
C24—C25—H25A	118.8	C54—C55—H55A	118.8
C25—C26—C27	121.5 (3)	C55—C56—C57	122.6 (3)
C25—C26—H26A	119.3	C55—C56—H56A	118.7
C27—C26—H26A	119.3	C57—C56—H56A	118.7
C18—C27—C28	115.2 (3)	C48—C57—C58	114.7 (2)
C18—C27—C26	115.3 (3)	C48—C57—C56	114.7 (2)
C28—C27—C26	129.5 (3)	C58—C57—C56	130.6 (3)
C29—C28—C27	120.4 (3)	C59—C58—C57	120.4 (3)
C29—C28—H28A	119.8	C59—C58—H58A	119.8
C27—C28—H28A	119.8	C57—C58—H58A	119.8
C28—C29—C30	123.2 (3)	C58—C59—C60	122.4 (3)
C28—C29—H29A	118.4	C58—C59—H59A	118.8
C30—C29—H29A	118.4	C60—C59—H59A	118.8
C17—C30—C29	117.7 (3)	C47—C60—C59	118.4 (2)
C17—C30—H30A	121.2	C47—C60—H60A	120.8
C29—C30—H30A	121.2	C59—C60—H60A	120.8
			
O1—C1—C2—C15	1.6 (5)	O1—C1—C2—C15	1.6 (5)
C5—C1—C2—C15	−177.9 (3)	C5—C1—C2—C15	−177.9 (3)
O1—C1—C2—C3	179.8 (3)	O1—C1—C2—C3	179.8 (3)
C5—C1—C2—C3	0.4 (3)	C5—C1—C2—C3	0.4 (3)
C15—C2—C3—C12	0.0 (4)	C15—C2—C3—C12	0.0 (4)
C1—C2—C3—C12	−178.7 (3)	C1—C2—C3—C12	−178.7 (3)
C15—C2—C3—C4	178.5 (2)	C15—C2—C3—C4	178.5 (2)
C1—C2—C3—C4	−0.1 (3)	C1—C2—C3—C4	−0.1 (3)
C12—C3—C4—C9	0.3 (4)	C12—C3—C4—C9	0.3 (4)
C2—C3—C4—C9	−178.3 (3)	C2—C3—C4—C9	−178.3 (3)
C12—C3—C4—C5	178.4 (3)	C12—C3—C4—C5	178.4 (3)
C2—C3—C4—C5	−0.2 (3)	C2—C3—C4—C5	−0.2 (3)
C9—C4—C5—C6	−1.2 (4)	C9—C4—C5—C6	−1.2 (4)
C3—C4—C5—C6	−179.2 (2)	C3—C4—C5—C6	−179.2 (2)
C9—C4—C5—C1	178.5 (3)	C9—C4—C5—C1	178.5 (3)
C3—C4—C5—C1	0.4 (3)	C3—C4—C5—C1	0.4 (3)
O1—C1—C5—C6	−0.4 (5)	O1—C1—C5—C6	−0.4 (5)
C2—C1—C5—C6	179.1 (3)	C2—C1—C5—C6	179.1 (3)
O1—C1—C5—C4	−180.0 (3)	O1—C1—C5—C4	−180.0 (3)
C2—C1—C5—C4	−0.5 (3)	C2—C1—C5—C4	−0.5 (3)
C4—C5—C6—C7	−0.2 (4)	C4—C5—C6—C7	−0.2 (4)
C1—C5—C6—C7	−179.7 (3)	C1—C5—C6—C7	−179.7 (3)
C5—C6—C7—C8	0.7 (4)	C5—C6—C7—C8	0.7 (4)
C6—C7—C8—C9	0.1 (5)	C6—C7—C8—C9	0.1 (5)
C5—C4—C9—C8	1.9 (4)	C5—C4—C9—C8	1.9 (4)
C3—C4—C9—C8	179.7 (2)	C3—C4—C9—C8	179.7 (2)
C5—C4—C9—C10	−178.0 (3)	C5—C4—C9—C10	−178.0 (3)
C3—C4—C9—C10	−0.2 (4)	C3—C4—C9—C10	−0.2 (4)
C7—C8—C9—C4	−1.3 (4)	C7—C8—C9—C4	−1.3 (4)
C7—C8—C9—C10	178.6 (3)	C7—C8—C9—C10	178.6 (3)
C4—C9—C10—C11	−0.1 (4)	C4—C9—C10—C11	−0.1 (4)
C8—C9—C10—C11	180.0 (3)	C8—C9—C10—C11	180.0 (3)
C9—C10—C11—C12	0.3 (5)	C9—C10—C11—C12	0.3 (5)
C2—C3—C12—C13	0.5 (4)	C2—C3—C12—C13	0.5 (4)
C4—C3—C12—C13	−177.9 (2)	C4—C3—C12—C13	−177.9 (2)
C2—C3—C12—C11	178.3 (3)	C2—C3—C12—C11	178.3 (3)
C4—C3—C12—C11	−0.1 (4)	C4—C3—C12—C11	−0.1 (4)
C10—C11—C12—C3	−0.3 (4)	C10—C11—C12—C3	−0.3 (4)
C10—C11—C12—C13	177.2 (3)	C10—C11—C12—C13	177.2 (3)
C3—C12—C13—C14	−0.9 (4)	C3—C12—C13—C14	−0.9 (4)
C11—C12—C13—C14	−178.3 (3)	C11—C12—C13—C14	−178.3 (3)
C12—C13—C14—C15	0.9 (5)	C12—C13—C14—C15	0.9 (5)
C3—C2—C15—C14	0.0 (4)	C3—C2—C15—C14	0.0 (4)
C1—C2—C15—C14	178.1 (3)	C1—C2—C15—C14	178.1 (3)
C13—C14—C15—C2	−0.4 (5)	C13—C14—C15—C2	−0.4 (5)
O2—C16—C17—C30	0.9 (6)	O2—C16—C17—C30	0.9 (6)
C20—C16—C17—C30	−179.0 (3)	C20—C16—C17—C30	−179.0 (3)
O2—C16—C17—C18	−179.8 (3)	O2—C16—C17—C18	−179.8 (3)
C20—C16—C17—C18	0.4 (3)	C20—C16—C17—C18	0.4 (3)
C30—C17—C18—C27	0.5 (4)	C30—C17—C18—C27	0.5 (4)
C16—C17—C18—C27	−179.0 (3)	C16—C17—C18—C27	−179.0 (3)
C30—C17—C18—C19	179.4 (3)	C30—C17—C18—C19	179.4 (3)
C16—C17—C18—C19	−0.1 (3)	C16—C17—C18—C19	−0.1 (3)
C27—C18—C19—C24	−0.3 (4)	C27—C18—C19—C24	−0.3 (4)
C17—C18—C19—C24	−179.2 (2)	C17—C18—C19—C24	−179.2 (2)
C27—C18—C19—C20	178.7 (2)	C27—C18—C19—C20	178.7 (2)
C17—C18—C19—C20	−0.3 (3)	C17—C18—C19—C20	−0.3 (3)
C24—C19—C20—C21	1.6 (4)	C24—C19—C20—C21	1.6 (4)
C18—C19—C20—C21	−177.3 (3)	C18—C19—C20—C21	−177.3 (3)
C24—C19—C20—C16	179.4 (3)	C24—C19—C20—C16	179.4 (3)
C18—C19—C20—C16	0.5 (3)	C18—C19—C20—C16	0.5 (3)
O2—C16—C20—C21	−3.4 (6)	O2—C16—C20—C21	−3.4 (6)
C17—C16—C20—C21	176.4 (4)	C17—C16—C20—C21	176.4 (4)
O2—C16—C20—C19	179.6 (3)	O2—C16—C20—C19	179.6 (3)
C17—C16—C20—C19	−0.5 (3)	C17—C16—C20—C19	−0.5 (3)
C19—C20—C21—C22	−0.6 (5)	C20—C19—C24—C23	−1.1 (4)
C16—C20—C21—C22	−177.3 (3)	C18—C19—C24—C23	177.7 (2)
C20—C21—C22—C23	−0.9 (5)	C20—C19—C24—C25	−179.8 (3)
C21—C22—C23—C24	1.4 (5)	C18—C19—C24—C25	−1.0 (4)
C20—C19—C24—C23	−1.1 (4)	C22—C23—C24—C19	−0.4 (4)
C18—C19—C24—C23	177.7 (2)	C22—C23—C24—C25	178.1 (3)
C20—C19—C24—C25	−179.8 (3)	C19—C24—C25—C26	1.5 (4)
C18—C19—C24—C25	−1.0 (4)	C23—C24—C25—C26	−177.0 (3)
C22—C23—C24—C19	−0.4 (4)	C24—C25—C26—C27	−0.8 (4)
C22—C23—C24—C25	178.1 (3)	C17—C18—C27—C28	0.3 (4)
C19—C24—C25—C26	1.5 (4)	C19—C18—C27—C28	−178.5 (2)
C23—C24—C25—C26	−177.0 (3)	C17—C18—C27—C26	179.7 (2)
C24—C25—C26—C27	−0.8 (4)	C19—C18—C27—C26	1.0 (4)
C17—C18—C27—C28	0.3 (4)	C25—C26—C27—C18	−0.4 (4)
C19—C18—C27—C28	−178.5 (2)	C25—C26—C27—C28	178.9 (3)
C17—C18—C27—C26	179.7 (2)	C18—C27—C28—C29	−0.4 (4)
C19—C18—C27—C26	1.0 (4)	C26—C27—C28—C29	−179.8 (3)
C25—C26—C27—C18	−0.4 (4)	C27—C28—C29—C30	−0.3 (5)
C25—C26—C27—C28	178.9 (3)	C18—C17—C30—C29	−1.2 (4)
C18—C27—C28—C29	−0.4 (4)	C16—C17—C30—C29	178.2 (3)
C26—C27—C28—C29	−179.8 (3)	C28—C29—C30—C17	1.1 (5)
C27—C28—C29—C30	−0.3 (5)	O3—C31—C32—C45	1.1 (5)
C18—C17—C30—C29	−1.2 (4)	C35—C31—C32—C45	−178.9 (3)
C16—C17—C30—C29	178.2 (3)	O3—C31—C32—C33	−179.7 (3)
C28—C29—C30—C17	1.1 (5)	C35—C31—C32—C33	0.3 (3)
O3—C31—C32—C45	1.1 (5)	C45—C32—C33—C42	0.2 (4)
C35—C31—C32—C45	−178.9 (3)	C31—C32—C33—C42	−179.2 (2)
O3—C31—C32—C33	−179.7 (3)	C45—C32—C33—C34	179.4 (2)
C35—C31—C32—C33	0.3 (3)	C31—C32—C33—C34	0.0 (3)
C45—C32—C33—C42	0.2 (4)	C42—C33—C34—C39	−0.7 (4)
C31—C32—C33—C42	−179.2 (2)	C32—C33—C34—C39	−180.0 (2)
C45—C32—C33—C34	179.4 (2)	C42—C33—C34—C35	178.8 (2)
C31—C32—C33—C34	0.0 (3)	C32—C33—C34—C35	−0.4 (3)
C42—C33—C34—C39	−0.7 (4)	C39—C34—C35—C36	0.3 (4)
C32—C33—C34—C39	−180.0 (2)	C33—C34—C35—C36	−179.2 (2)
C42—C33—C34—C35	178.8 (2)	C39—C34—C35—C31	−179.9 (2)
C32—C33—C34—C35	−0.4 (3)	C33—C34—C35—C31	0.6 (3)
C39—C34—C35—C36	0.3 (4)	O3—C31—C35—C36	−0.8 (5)
C33—C34—C35—C36	−179.2 (2)	C32—C31—C35—C36	179.2 (3)
C39—C34—C35—C31	−179.9 (2)	O3—C31—C35—C34	179.4 (3)
C33—C34—C35—C31	0.6 (3)	C32—C31—C35—C34	−0.6 (3)
O3—C31—C35—C36	−0.8 (5)	C34—C35—C36—C37	−0.7 (4)
C32—C31—C35—C36	179.2 (3)	C31—C35—C36—C37	179.5 (3)
O3—C31—C35—C34	179.4 (3)	C35—C36—C37—C38	0.3 (4)
C32—C31—C35—C34	−0.6 (3)	C36—C37—C38—C39	0.6 (4)
C34—C35—C36—C37	−0.7 (4)	C35—C34—C39—C38	0.6 (4)
C31—C35—C36—C37	179.5 (3)	C33—C34—C39—C38	−180.0 (2)
C35—C36—C37—C38	0.3 (4)	C35—C34—C39—C40	−179.5 (2)
C36—C37—C38—C39	0.6 (4)	C33—C34—C39—C40	−0.1 (4)
C35—C34—C39—C38	0.6 (4)	C37—C38—C39—C34	−1.0 (4)
C33—C34—C39—C38	−180.0 (2)	C37—C38—C39—C40	179.2 (3)
C35—C34—C39—C40	−179.5 (2)	C34—C39—C40—C41	0.7 (4)
C33—C34—C39—C40	−0.1 (4)	C38—C39—C40—C41	−179.4 (3)
C37—C38—C39—C34	−1.0 (4)	C39—C40—C41—C42	−0.5 (4)
C37—C38—C39—C40	179.2 (3)	C32—C33—C42—C43	−0.3 (4)
C34—C39—C40—C41	0.7 (4)	C34—C33—C42—C43	−179.4 (2)
C38—C39—C40—C41	−179.4 (3)	C32—C33—C42—C41	180.0 (2)
C39—C40—C41—C42	−0.5 (4)	C34—C33—C42—C41	0.9 (4)
C32—C33—C42—C43	−0.3 (4)	C40—C41—C42—C33	−0.3 (4)
C34—C33—C42—C43	−179.4 (2)	C40—C41—C42—C43	−179.9 (3)
C32—C33—C42—C41	180.0 (2)	C33—C42—C43—C44	0.2 (4)
C34—C33—C42—C41	0.9 (4)	C41—C42—C43—C44	179.8 (3)
C40—C41—C42—C33	−0.3 (4)	C42—C43—C44—C45	0.1 (4)
C40—C41—C42—C43	−179.9 (3)	C33—C32—C45—C44	0.0 (4)
C33—C42—C43—C44	0.2 (4)	C31—C32—C45—C44	179.1 (3)
C41—C42—C43—C44	179.8 (3)	C43—C44—C45—C32	−0.1 (4)
C42—C43—C44—C45	0.1 (4)	O4—C46—C47—C60	−2.2 (5)
C33—C32—C45—C44	0.0 (4)	C50—C46—C47—C60	177.1 (3)
C31—C32—C45—C44	179.1 (3)	O4—C46—C47—C48	−179.6 (2)
C43—C44—C45—C32	−0.1 (4)	C50—C46—C47—C48	−0.3 (3)
O4—C46—C47—C60	−2.2 (5)	C60—C47—C48—C57	−0.5 (4)
C50—C46—C47—C60	177.1 (3)	C46—C47—C48—C57	177.5 (2)
O4—C46—C47—C48	−179.6 (2)	C60—C47—C48—C49	−177.9 (2)
C50—C46—C47—C48	−0.3 (3)	C46—C47—C48—C49	0.1 (3)
C60—C47—C48—C57	−0.5 (4)	C57—C48—C49—C54	0.2 (4)
C46—C47—C48—C57	177.5 (2)	C47—C48—C49—C54	177.7 (2)
C60—C47—C48—C49	−177.9 (2)	C57—C48—C49—C50	−177.3 (2)
C46—C47—C48—C49	0.1 (3)	C47—C48—C49—C50	0.2 (3)
C57—C48—C49—C54	0.2 (4)	C54—C49—C50—C51	−0.1 (4)
C47—C48—C49—C54	177.7 (2)	C48—C49—C50—C51	177.3 (2)
C57—C48—C49—C50	−177.3 (2)	C54—C49—C50—C46	−177.8 (2)
C47—C48—C49—C50	0.2 (3)	C48—C49—C50—C46	−0.4 (3)
C54—C49—C50—C51	−0.1 (4)	O4—C46—C50—C51	2.6 (5)
C48—C49—C50—C51	177.3 (2)	C47—C46—C50—C51	−176.7 (3)
C54—C49—C50—C46	−177.8 (2)	O4—C46—C50—C49	179.8 (2)
C48—C49—C50—C46	−0.4 (3)	C47—C46—C50—C49	0.5 (3)
O4—C46—C50—C51	2.6 (5)	C49—C50—C51—C52	0.6 (4)
C47—C46—C50—C51	−176.7 (3)	C46—C50—C51—C52	177.6 (3)
O4—C46—C50—C49	179.8 (2)	C50—C51—C52—C53	−0.8 (4)
C47—C46—C50—C49	0.5 (3)	C51—C52—C53—C54	0.3 (4)
C49—C50—C51—C52	0.6 (4)	C50—C49—C54—C53	−0.4 (4)
C46—C50—C51—C52	177.6 (3)	C48—C49—C54—C53	−177.5 (2)
C50—C51—C52—C53	−0.8 (4)	C50—C49—C54—C55	178.1 (2)
C51—C52—C53—C54	0.3 (4)	C48—C49—C54—C55	1.0 (3)
C50—C49—C54—C53	−0.4 (4)	C52—C53—C54—C49	0.2 (4)
C48—C49—C54—C53	−177.5 (2)	C52—C53—C54—C55	−177.9 (2)
C50—C49—C54—C55	178.1 (2)	C49—C54—C55—C56	−0.9 (4)
C48—C49—C54—C55	1.0 (3)	C53—C54—C55—C56	177.2 (3)
C52—C53—C54—C49	0.2 (4)	C54—C55—C56—C57	−0.3 (4)
C52—C53—C54—C55	−177.9 (2)	C47—C48—C57—C58	0.6 (4)
C49—C54—C55—C56	−0.9 (4)	C49—C48—C57—C58	177.7 (2)
C53—C54—C55—C56	177.2 (3)	C47—C48—C57—C56	−178.5 (2)
C54—C55—C56—C57	−0.3 (4)	C49—C48—C57—C56	−1.4 (3)
C47—C48—C57—C58	0.6 (4)	C55—C56—C57—C48	1.4 (4)
C49—C48—C57—C58	177.7 (2)	C55—C56—C57—C58	−177.4 (3)
C47—C48—C57—C56	−178.5 (2)	C48—C57—C58—C59	−0.2 (3)
C49—C48—C57—C56	−1.4 (3)	C56—C57—C58—C59	178.7 (2)
C55—C56—C57—C48	1.4 (4)	C57—C58—C59—C60	−0.3 (4)
C55—C56—C57—C58	−177.4 (3)	C48—C47—C60—C59	0.0 (4)
C48—C57—C58—C59	−0.2 (3)	C46—C47—C60—C59	−177.2 (3)
C56—C57—C58—C59	178.7 (2)	C58—C59—C60—C47	0.4 (4)
C57—C58—C59—C60	−0.3 (4)	C24—C23—C22—C21	1.4 (5)
C48—C47—C60—C59	0.0 (4)	C19—C20—C21—C22	−0.6 (5)
C46—C47—C60—C59	−177.2 (3)	C16—C20—C21—C22	−177.3 (3)
C58—C59—C60—C47	0.4 (4)	C23—C22—C21—C20	−0.9 (5)
